# Supplementary material for: Automated segmentation and quantitative analysis of organelle morphology, localization and content using CellProfiler
Source: PLoS One. 2023 Jun 14;18(6):e0278009. doi: 10.1371/journal.pone.0278009 (PMC10266606; doi:10.1371/journal.pone.0278009)
Supplement: S1 Table — (DOCX) [file pone.0278009.s001.docx]

**Supplemental Table 1. Antibodies used in immunofluorescence (IF)**

| **Antibody** | **Manufacturer** | **Cat. Number** | **Dilution** |
| --- | --- | --- | --- |
| IF Primary (OP) | | | |
| VWF (rabbit) | DAKO | A0082 | 1:1000/ 1:50000 |
| VE-cadherin (mouse) | BD Pharming | 55561 | 1:250 |
| β-catenin (rabbit) | Santa Cruz | Sc-7199 | 1:500 |
| EEA1 (mouse) | BD Biosciences | 610457 | 1:500 |
| Hoechst | Sigma-Aldrich | H3569 | 1:10000 |
| DAPI | Thermo Fisher | D3571 | 1:33000 |
| VE-cadherin (goat) | R&D systems | AF938 | 1:250 |
| VE-cadherin (mouse) | Santa Cruz | sc-9989 | 1:250 |
| TGN46 (sheep) | Serotec | AHP500 | 1:1000 |
| IF Primary (OCP) | | | |
| VWF (sheep) | Abcam | ab11713 | 1:1000 |
| VE-cadherin (mouse) | BD Pharming | 55561 | 1:250 |
| Hoechst | Sigma-Aldrich | H3569 | 1:10000 |
| Rab27A (rabbit) | Protein Tech | 17817 | 1:100 |
| PDI (rabbit) | Enzo Life Sciences | SPA-890 | 1:250 |
| IF Secondary | | | |
| Donkey-anti-Rabbit AF647 | Invitrogen Molecular Probes | A31573 | 1:750 |
| Donkey-anti-Mouse AF568 | Invitrogen Molecular Probes | A10037 | 1:750 |
| Donkey-anti-Sheep AF488 | Invitrogen Molecular Probes | A11015 | 1:750 |
| Donkey-anti Rabbit AF568 | Invitrogen Molecular Probes | A10042 | 1:400 |
| Donkey-anti Goat AF647 | Invitrogen Molecular Probes | A32849 | 1:400 |
| Donkey-anti Mouse CF568 | Biotium | 20105 | 1:1000 |
| Donkey-anti Mouse CF488A | Biotium | 20014 | 1:1000 |
