# Supplementary material for: Automated segmentation and quantitative analysis of organelle morphology, localization and content using CellProfiler
Source: PLoS One. 2023 Jun 14;18(6):e0278009. doi: 10.1371/journal.pone.0278009 (PMC10266606; doi:10.1371/journal.pone.0278009)
Supplement: S7 File — (DOCX) [file pone.0278009.s008.docx]

**Example Pipeline OrganelleProfiler - Identifying and quantifying nuclei, cells and organelles**

Here, each module of the OrganelleProfiler and OrganelleContentProfiler is explained. Endothelial Colony Forming Cells and Weibel-Palade Bodies are used as main example of the settings. However, for some modules additional information is supplied how modules were adjusted for different applications.

**1. input of Images**

The images are dropped or uploaded in the “drop files and folder here” box. Images should have 3 channels; a nuclei staining (here: Hoechst), a cell membrane staining (VE-cadherin) and an organelle specific staining (VWF) for the pipeline to work. Under “NamesAndTypes” the image type “Color image” is selected and named “Original”.

**2. ColorToGray**

Here, the 3 channels are separated and identified as individual gray channels. The input is the “Original” image defined in step 1. The conversion method is “split” the channels need to be separated. Channels should be named accurately as the names are used later in the pipeline and are case sensitive. In this example the channels are named “Nuclei”, “Cell_Membrane” and “Organelle”

**3. Smooth**

The pipeline starts by identifying the Nuclei. In the Smooth module the “Nuclei” gray channel is chosen as input and named “Smoothed_Nuclei”. This smooths the objects removing small Imaging artefacts, noise and other non-nuclear structures stained by the Hoechst dye, making the later object identification more accurate.

**4. Threshold**

The “Smoothed_Nuclei” is used as input and the output is named “Threshold_Nuclei”. Using a Global threshold strategy and the Minimum Cross-Entropy method a binary (black and white) version of the grayscale image is created. This binary image yields better nuclei identification. The variables are optimized for the images to accurately retain the shape of the nuclei. If the binary image does not accurately represent the nuclei then these variables have to be altered.

**5. IdentifyPrimaryObjects (Nuclei)**

This module uses the “Threshold_Nuclei” as input for the identification of the nuclei as objects which are named “Nuclei_Object”. This module offers many options to tweak and optimize the identification of objects based on their size, shape, intensity and proximity to other objects. This module was optimized for nuclei with an approximate size of 200 pixels. Cell profiler image output windows have a scale that can be used to measure objects and adjust the threshold variables accordingly. As the signal from the nuclei is binary, all the variables are set to use the shape of the object for identification. Some cells have 2 nuclei, but these must not be identified as 2 cells. Therefore, the minimum allowed distance between local maxima was set to 100. This means that objects closer than 100 pixels to each other will be identified as one object.

**6. Smooth**

The second object that the pipeline identifies are the cells. For this the “Cell_Membrane” (CM) image is used as input which is smoothed similarly to the “Nuclei” channel in step 3. This is done to make the object identification more accurate. The output is named “CM_Smoothed”.

**7. IdentifySecondaryObjects (Cells)**

This module uses the “Nuclei_Object” as input object and the “CM_Smoothed” as input image to create a secondary object, the “Cells_Object”. The method used to identify the cells is propagation. In this method, the nucleus is the starting point of the object, which propagates outward until it reaches the CM signal. The output should be checked to confirm the cell membrane is accurately used to create the “Cells_Object”. If not, then the threshold strategy or variables have to be adjusted. Endothelial cells form a confluent layer in culture. Thus, there is no empty space in the images. For this reason the Threshold correction factor is set to 0. When analyzing non-confluent cell types, this module can be easily adjusted by increasing the Threshold correction factor. In the case of the HEK293T cells, the value was set to 0.2.

**8. IdentifyTertiaryObjects (Membrane)**

Using the “Cells_Object” made in step 7, a cell membrane object named “CM_Object” can be generated. This is needed later in the pipeline to relate objects within the cell membrane (step 6 in the OCP pipeline). “Cells_Object” is selected for both the larger and smaller identified objects and the smaller object is shrunk. The result of this is a 1 pixel thick line of the cell membrane that can be used to separate signal between cells.

**9. RescaleIntensity**

The final object to be identified is the organelle, in this example the WPB. In this module the “Organelle” channel is the input and the output is named “Rescale_Intensity_Organelle”. The rescaling method used is “Stretch each image to use the full intensity range”. This is done to rescale the VWF signal to make the object identification consistent over multiple samples.

**10. EnhanceOrSuppressFeatures 1 & 2**

In these two modules the “Rescale_Intensity_Organelle” signal is enhanced via 2 methods. First, to differentiate between Organelles that are lying close to or on top of each other, the operation “enhance” and “speckles” is chosen to create “Speckles_Organelle”. Second, to distinguish separate objects further, the operation “enhance”, “neurites” and “line structures” is chosen to create “Neurites_Organelle”. When applied to samples with elongated Organelles, this module will emphasize these structures. The effect on single organelles is minimal and does not influence shape significantly. Do note that these optimization steps increase the accuracy of the object identification, but they also affect VWF intensity levels. Intensity measurements should thus be performed on the raw input. When analyzing round organelles, like endosomes, the EnhanceOrSuppressFeatures module “neurites” should be turned off.

**11. IdentifyPrimaryObjects (Organelle)**

This module identifies the WPBs as objects (“Organelle_Object”) in a similar way to step 5. The input image is “Neurites_Organelle”. The threshold strategy, variables and other options are optimized for the recognition of objects roughly 15 pixels in size with a strong fluorescent signal.

**12. MeasureObjectIntensity & MeasureObjectSizeShape**

Measure size and measure intensity modules can be adjusted to add or remove measurements to fit the research question. Exporting additional measurements is easily done with these modules. Note that each measurement yields multiple variables per object type or type of measurement which significantly increases computing time.

**13. RelateObjects**

To identify which WPBs are located in which cells a child-parent relation is needed. The parent object is “Cells_Object” and the child object is “Organelle_Object”. An optional measurement is the child-parent distance. This will yield, in pixels, the distance from the center of the child object to the closest part of the parent objects. In this example we calculate the distance to the cell membrane using “Cells_Object” as parent and to the nuclei using “Nuclei_Object” as parent. Note that including distance measurements will increase computing time.

**14. OverlayOutlines**

During optimization of the pipeline it is important to check whether every module accurately processes the input. However, when the pipeline is optimized and images are analyzed in bulk it is not practical to continuously check the output. This module creates an overlay of all objects and projects them on “Rescale_Intensity_Organelle” made in step 9. The selected objects are “Cells_Object”, “Organelle_Object” and “Nuclei_Object”. This allows for easy quality control of all the key modules used up to this point. The output is named “Overlay”.

**15. DisplayDataOnImage**

The “Object_Numbers” of the “Cells_Object” are displayed on the “Overlay” image for further quality control. This image is named “Overlay_Numbered”. When exported data shows outliers, individual cells can now be easily identified. If these values are affected by some artefact they can then be excluded from analysis or the appropriate modules can be adjusted.

**16. SaveImages**

This module exports “Overlay_Numbered” made in step 14. PNG format is recommended as the saved file format because this format retains high enough resolution for the quality control of the objects and the file size is significantly smaller than the TIFF format.

**17. ExportToDatabase**

The final module exports the data to a database. The “ExportToDatabase” module is preferred over the “ExportToSpreadsheet” module as the database is updated while the pipeline is working and the spreadsheet is made only when the pipeline is done. When an error occurs halfway through a batch processing, the database will contain all completed samples. In this module you can also select which measurements you want to export.

**Example Pipeline OrganelleContentProfiler - Identifying and measuring other organelle proteins.**

**1. ColorToGray**

Here, an additional channel for the Rab27A staining “Organelle_Content (OC)” is added to the module described in the OrganelleProfiler pipeline step 2. All other settings remain the same.

**2. ExpandOrShrinkObjects – Optional**

The “Organelle_Object” was identified using the VWF protein, which is only present inside the WPB. Rab27A is present on the surface of the organelle and thus will give a bigger signal. To ensure complete encapsulation of the Rab27A signal, “Organelle_Object” was expanded by 2 pixels. The output is named “Expanded_Organelle_Object”.

**3. RescaleIntensity**

For the OverlayOutlines module, the input image has to be rescaled for the signal to be viewable with the eye. Therefore, the “Rab27A” intensity is rescaled in this module. The output is named “Rescale_Intensity_OC” and the method used is “Stretch each image to use the full intensity range”.

**4. MaskImage**

This module masks portions of an image based on previously identified objects. Here, the input image is “Rescale_Intensity_OC” and “Expanded_Organelle_Object” is used as the object to create the mask. In this module the mask is not inverted resulting in an image containing only the Rab27A signal within the WPBs. The output is named “OC_In_Organelle_Mask”.

**5. IdentifyPrimaryObjects (Staining inside organelle)**

The masked Rab27A signal inside the WPB is identified as objects (“OC_In_Organelle_Object”) in a similar way to step 11 of the OrganelleProfiler pipeline. The input image is “OC_In_Organelle_Mask”. The threshold strategy, variables and other options are optimized for the recognition of objects roughly 15 pixels in size with any intensity level. As Rab27A signal can be very low in WPBs the threshold is set to include all values within the object.

**6. MaskImage 2 + 3**

In these two modules, inverse masks are placed on the “OC” channel. First, the “Expanded_Organelle_Object” is used to isolate the Rab27A signal outside of the WPBs. The output is named “OC_Out_Organelle_Mask”. In the second masking module, with “OC_Out_Organelle_Mask” as input, the image is masked using the “CM_Object” (from the OP pipeline step 8). This separates the Rab27A signal outside of the WPBs per cell. The output is named “OC_Out_Organelle_Mask_Cell”. This step is needed for the identification of this signal per cell as an object in step 7.

**7. IdentifyPrimaryObjects (staining outside organelle)**

This module identifies the Rab27A signal outside the WPB as objects (“OC_Out_Organelle_Object”) in a similar way to step 4 of the OrganelleContentProfiler pipeline. The input image is “OC_Out_Organelle_Mask_Cell”. The threshold strategy, variables and other options are optimized for the recognition of objects roughly 1000 pixels in size with any intensity level.

**8. RelateObjects 1 + 2**

Similar to step 12 in the OrganelleProfiler pipeline, a child-parent relation is made between Rab27A and in which cell the objects are measured. The parent object on both modules is “Cells_Object” and the child object is “OC_In_Organelle_Object” and “OC_Out_Organelle_Object”.
